# Vehicle Lateral State Estimation Based on Measured Tyre Forces

**DOI:** 10.3390/s91108761

**Published:** 2009-10-30

**Authors:** Ari J. Tuononen

**Affiliations:** Laboratory of Automotive Engineering, Helsinki University of Technology, P.O. Box 4300, 02015 TKK, Finland; E-Mail: ari.tuononen@tkk.fi; Tel.: +358-50 5604702; Fax: +358-9 4513469

**Keywords:** optical position detection, intelligent tyre, tyre sensor, vehicle state estimation

## Abstract

Future active safety systems need more accurate information about the state of vehicles. This article proposes a method to evaluate the lateral state of a vehicle based on measured tyre forces. The tyre forces of two tyres are estimated from optically measured tyre carcass deflections and transmitted wirelessly to the vehicle body. The two remaining tyres are so-called virtual tyre sensors, the forces of which are calculated from the real tyre sensor estimates. The Kalman filter estimator for lateral vehicle state based on measured tyre forces is presented, together with a simple method to define adaptive measurement error covariance depending on the driving condition of the vehicle. The estimated yaw rate and lateral velocity are compared with the validation sensor measurements.

## 1. Introduction

The vehicle state estimation has been a subject for numerous papers, especially because of the large scale market penetration of Electronic Stability Control (ESC) systems. Even then, more accurate and reliable vehicle state information is needed for upcoming active control applications such as active steering (front and rear), lane keeping and torque vectoring.

The main disadvantage of most of the vehicle state estimation approaches is the requirement for prior knowledge of tyre parameters such as cornering stiffness and friction coefficient [1-4]. However, model based estimation is very accurate when these parameters are known. A fresh vehicle sideslip estimator approach is presented in [5], where a kinematic approach is implemented, using a 6-degree-of-freedom Inertial Measurement Unit (IMU). The estimator is independent of any troublesome parameters and any drifting of the estimate during low lateral excitation is avoided by implementing a Kalman Filter with an adaptive covariance matrix. The estimate based on kinematic formulae can also be supported by a vehicle state observer by weighting estimates depending on the driving state [6]. A similar estimation strategy is implemented here, but instead of measuring accelerations and rotational velocities of a vehicle body, the origin of those quantities, the tyre forces, are measured directly.

**Figure 1.**
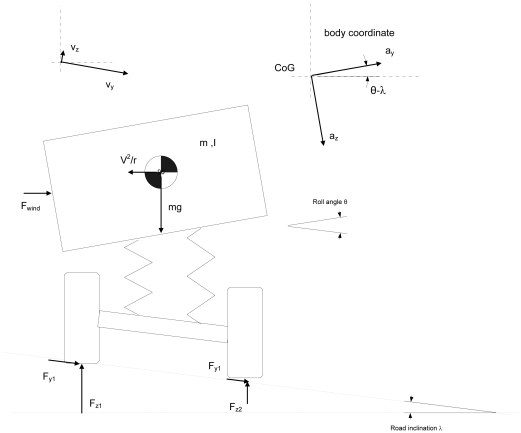
Lateral forces acting on one axle of cornering vehicle.

The advantage of tyre force measurement can be seen in Figure 1, where a vehicle is cornering under road inclination *λ*, side wind F_wind_ and roll angle *θ*. The tyre lateral tyre forces are parallel to lateral velocity v_y_. If the state estimation is based on lateral acceleration the following errors exist:

- roll angle θ and road inclination λ introduce offset for lateral acceleration a_y_ sensor due to gravity component (5,7° roll angle results 0.1g error for lateral acceleration)
  $$a_{y,\text{global}}=\frac{a_{y,\text{measured}}-g\sin(\theta -\lambda )}{\cos(\theta -\lambda )}$$
- Influence of side wind is not properly captured from the measured acceleration, but has to be carried by the tyres (and influence for v_y_ is missing)
- v_y_ is not even parallel to compensated lateral acceleration $v_{y}=\frac{\int \left(a_{y,\text{global}}-\dot{\psi }v_{x}\right)dt}{\cos\lambda }$

The roll angle and road inclination influence the yaw rate measurement as well, but contrary to the lateral acceleration, gravity does not participate and thus the overall impact is minor (the same applies for pitch angle).

Consequently, the direct measurement of tyre forces seems beneficial in contrast to body accelerations and rotational velocities. Possible technologies for the tyre force measurement could be:

- strain measurement of suspension components [7] or rim [8]
- measurement of tyre carcass displacement [9,10], acceleration [e.g.^11^] or strain [11]
- tyre tread displacement [12]
- force sensing bearing [13]

Even though there have been many attempts to measure tyre forces to aid control systems, there are very few articles which study how to exploit them [8,14,15]. This paper proposes a Kalman Filter estimation for vehicle yaw rate *ψ̇* and lateral velocity v_y_ based on measured tyre forces.

## 2. Optical Tyre Sensor (OTS) Concept

The optical tyre sensor was developed for the first time in the EC-funded APOLLO-project [16], which studied several different tyre sensor concepts, including the optical tyre sensor (OTS). The APOLLO was followed by the FRICTI@N-project, where the optical tyre sensor was selected for further development. During FRICTI@N, tyre sensor hardware was refreshed and algorithms were made more robust and towards real-time operation. A truck sensor was also introduced [17]. The sensor was also tested under aquaplaning conditions, where the transition point from the hydrodynamic aquaplaning zone to the viscous aquaplaning was measured [18]. In addition, the severity of aquaplaning was estimated in real-time [19].

**Figure 2.**
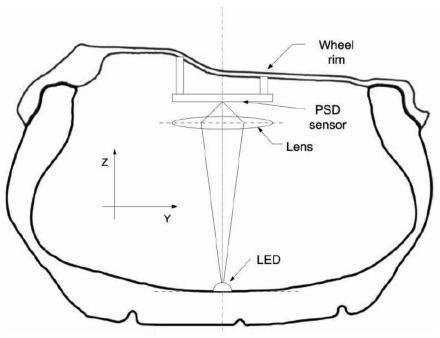
Optical tyre sensor measurement principle [10].

The OTS measuring principle is shown in Figure 2. A Light Emitting Diode (LED) is glued into the inner liner of the tyre. A lens focuses infrared light emitted by the LED to the surface of Position Sensitive Detector (PSD). The position of the light spot is relative to the current at the corners of the PSD. The raw data is analysed in the tyre and sent wirelessly by radio at 433MHz to a receiver unit. A more detailed explanation about OTS can be found in [10,17]. The algorithms for the tyre force estimation can be found in [17].

**Figure 3.**
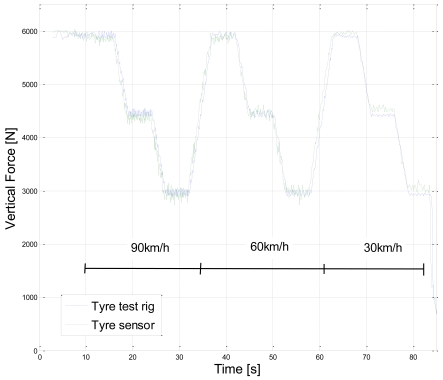
Tyre sensor calibration cycle for vertical force and comparison with test rig measurement.

**Figure 4.**
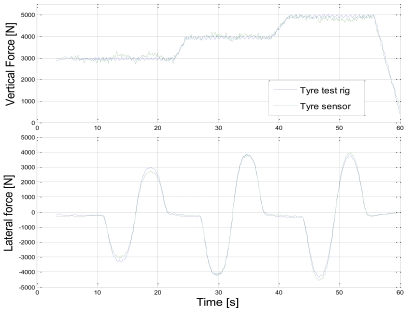
Tyre sensor calibration cycle for lateral force and comparison with test rig measurement (vertical force and lateral forces during cycle, 60 km/h).

Figure 3 shows the calibration cycle for vertical force and tyre sensor measurement compared with the test rig results. Three different wheel loads are varied at different speeds. Similarly, Figure 4 shows the calibration cycle for the lateral force at three different loads. The tyre force estimation model parameters are fitted to test rig results by the least squares method.

## 3. Vehicle State Estimator

**Figure 5.**
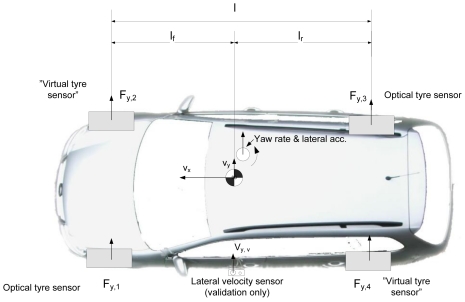
Measurement car setup.

A test car setup can be seen in Figure 5. The optical tyre sensors are mounted at the left front wheel and rear right wheel position. In addition, the steering wheel angle and vehicle velocity (from the CAN-bus) are used in the estimation. All the other sensors, such as lateral acceleration, yaw rate and Correvit-sensor are for validation purposes only. These sensors are mounted close to the centre of gravity to avoid compensations.

**Figure 6.**
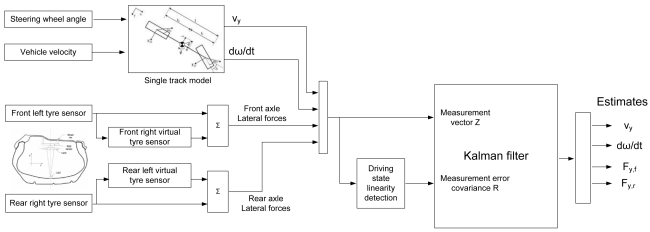
Block diagram of the estimator.

The problem is to estimate lateral velocity and yaw rate at the centre of gravity of the vehicle with a minimal set of parameters. The estimator consists of virtual tyre sensors, a driving state estimator (linearity) and a Kalman filter. The overall structure of the estimator is shown in Figure 6. The operation of the submodels is explained in the following.

### 3.1. Kalman filter

The Kalman filter is an effective and recursive solution for the discrete data filtering problem from noisy measurements [20]. The state transition reads:

(1) $$x_{k}=Ax_{k-1}+w_{k-1}$$

and with measurement z_k_:

(2) $$z_{k}=Hx_{k}+v_{k}$$

where A is state transition matrix, H is measurement matrix, and w_k_ and v_k_ represent process and measurement noise. The *a priori* ${\hat{x}}_{k}^{-}$ (based on process knowledge) estimate error is:

(3) $$e_{k}^{-}=x_{k}-{\hat{x}}_{k}^{-}$$

and *a posteriori* (based on given measurement z_k_) estimate error is:

(4) $$e_{k}=x_{k}-{\hat{x}}_{k}$$

The *a priori* estimate error covariance is:

(5) $$P_{k}^{-}=E\left[e_{k}^{-}e_{k}^{-T}\right]$$

and the *a posteriori* estimate error covariance is:

(6) $$P_{k}=E\left[e_{k}{\text{e}}_{k}^{T}\right]$$

and Kalman gain:

(7) $$K=P_{k}^{-}H^{T}\;{\left(HP_{k}^{-}H^{T}+R\right)}^{-1}$$

which minimizes the *a posteriori* estimate error covariance [21].

The Kalman gain weights the *a priori* estimate and residual $z_{k}-H{\hat{x}}_{k}^{-}$:

(8) $${\hat{x}}_{k}={\hat{x}}_{k}^{-}+K\left(z_{k}-H{\hat{x}}_{k}^{-}\right)$$

The Kalman filter expects the process and measurements noise to be with normal probability distribution:

(9) p(w)∼N(0,Q)

(10) p(v)∼N(0,R)

where Q is process noise covariance and R is measurement noise covariance matrix. In this paper, this requirement for normal probability distribution is not fulfilled all the time for all measurements; this it is discussed in more detail in section 3.5.

### 3.2. Virtual tyre sensors

The test car was equipped with two optical tyre sensors. The sensor positions were at the left front and right rear wheels. However, the vehicle state estimator developed here requires individual tyre forces of all tyres or axle forces. Thus, the lateral tyre forces of right front wheel and left rear wheel have to be estimated. The natural way to estimate is to exploit the information from the tyre sensor at the same axle (only lateral dynamics considered here). Hence, the “similarity method” [e.g., 22] is assumed in order to estimate the missing tyre forces. There are several methods to do this, two of which are presented here.

#### 3.2.1. Inverse magic formula

The obvious starting point is to solve slip angle α of the tyre from tyre sensor forces F_y_ and F_z_. The simple four parameter Magic Formula reads [22]:

(11) $$F_{y}=D\sin(C\;a\tan(B\;\alpha -E(B\;\alpha -a\tan(B\alpha ))))$$

where parameters B, C and E are assumed to be known. The D is available from the measured wheel load, and the slip angle α can be solved numerically.

Another required variable for a virtual tyre sensor is vertical load. The wheel load deviation of a tyre sensor wheel is available:

(12) $$\Delta F_{z,1}=F_{z,1}-F_{z,1,\text{static}}$$

where static wheel load can be calculated from the long time average of measured F_z,1_ or by recording F_z,1_ values as a static wheel load when F_y,1_ ∼ 0. The vertical load of the virtual tyre sensor (neglecting longitudinal load transfer and mass of vehicle is constant):

(13) $$F_{z,2}=F_{z,2,\text{static}}-\Delta F_{z,1}$$

The lateral force of the virtual tyre sensor can be then calculated with the same Equation 11 as when the slip angle was solved. It should be noted that the method is not sensitive for parameters B, C, or E if they do not depend on wheel load and if D is linear to wheel load. This is in fact an expression of a similarity method.

#### 3.2.2. Normalised lateral force

When considering the inverse magic formula method to solve the lateral force of the second tyre, it is clear that there has to be simpler method to obtain it. The normalized lateral force reads:

(14) $$\mu_{f}=\frac{F_{y,1}}{F_{z,1}}$$

and the lateral force of the second tyre at the same axle:

(15) $$F_{y,2}=\mu_{f}\;F_{z,2}$$

The vertical load F_z,2_ of the virtual tyre sensor is calculated as in Equation 13. The lateral force for the left rear tyre is calculated similarly to that at the front axle. The normalised lateral force method results in the same behaviour as the inverse magic formula. However, neither of the methods in this form takes into account normalised tyre force non-linearity for high wheel loads.

### 3.3. Vehicle model for the estimator

The Kalman filter requires a state transition matrix (without linearity assumption for tyre behaviour), which is here based on the lateral and yaw equations of motion:

(16) F=ma

(17) $$I_{z}\ddot{\psi }=F_{y,f}\;l_{f}-F_{y,r}\;l_{r}$$

where m is vehicle mass, I_z_ is yaw moment of inertia, l_f_ and l_r_ are centre of gravity distances from the front and rear axles. The lateral equation can be written:

(18) $$F_{y,f}+F_{y,r}=m\left({\dot{v}}_{y}+\dot{\psi }v_{x}\right)$$

where *F_y,f_* and *F_y,r_* are the front and rear axle forces. The acceleration of lateral motion can be solved:

(19) $${\dot{v}}_{y}=\frac{F_{y,f}+F_{y,r}}{m}-\dot{\psi }v_{x}$$

Lateral tyre forces are assumed to act on the centre of the contact patch (neglecting pneumatic trail), thus the yaw acceleration can be expressed:

(20) $$\ddot{\psi }=\frac{F_{y,f}\;l_{f}}{I_{z}}-\frac{F_{y,r}\;l_{r}}{I_{z}}$$

The Equations 19 and 20 are discretized and written as the state space equation:

(21) $$\left[\begin{array}{c} v_{y}(k+1)\\ \dot{\psi }(k+1)\\ F_{y,f}(k+1)\\ F_{y,r}(k+1) \end{array}\right]=\left\lfloor \begin{array}{cccc} 1 & -T_{s}v_{x} & \frac{T_{s}}{m} & \frac{T_{s}}{m}\\ 0 & 1 & \frac{l_{f}}{I_{z}}T_{s} & -\frac{l_{r}}{I_{z}}T_{s}\\ 0 & 0 & 1 & 0\\ 0 & 0 & 0 & 1 \end{array}\right\rfloor \;\left[\begin{array}{c} v_{y}(k)\\ \dot{\psi }(k)\\ F_{y,f}(k)\\ F_{y,r}(k) \end{array}\right]$$

where the only variable is forward velocity v_x_, which is assumed to be slowly changing. T_s_ is time step. The tyre force state transition is modelled as an identity function. The measurement matrix reads:

(22) $$y=\left[\begin{array}{cccc} 1 & 0 & 0 & 0\\ 0 & 1 & 0 & 0\\ 0 & 0 & 1 & 0\\ 0 & 0 & 0 & 1 \end{array}\right]\;\left[\begin{array}{c} v_{y}(k)\\ \dot{\psi }(k)\\ F_{y,f}(k)\\ F_{y,r}(k) \end{array}\right]$$

### 3.4. Single track model for the linear operation region

A simple vehicle model is needed to provide vehicle yaw rate, lateral velocity and tyre forces for the other subsystems of the estimator. The single track (or bicycle model) [e.g., 23] is based on lateral and yaw equations of motion:

(23) $$m\left({\dot{v}}_{y}+v_{x}\dot{\psi }\right)=F_{f}+F_{r}$$

(24) $$I\ddot{\psi }=l_{1}F_{1}-l_{2}F_{2}$$

The axle forces F_f_ and F_r_ are assumed to be linear to the slip angle:

(25) $$F_{f}=C_{f}\;\alpha_{f}$$

(26) $$F_{r}=C_{r}\;\alpha_{r}$$

where C_f_ and C_r_ are the front and rear axle cornering stiffness. The slip angles for the front and rear axle read:

(27) $$\alpha_{f}=\delta -\frac{v_{y}+l_{f}\dot{\psi }}{v_{x}}$$

(28) $$\alpha_{r}=\frac{l_{r}\dot{\psi }-v_{y}}{v_{x}}$$

where δ is steering angle at wheel.

### 3.5. Covariance matrixes for Kalman filter

#### 3.5.1. Process noise covariance matrix

The process noise variance matrix Q is assumed to be constant with very high process variance for tyre forces because a new measurement for tyre forces is always more accurate than *a priori* estimate, due to identity state transition in Equation 21. The suitable process variance for the lateral motion v_y_ and *ψ̇* is not of importance as long it has a realistic scale compared to the corresponding measurement noise variance to allow a tyre force based estimation during non-linear operation.

#### 3.5.2. Measurement noise covariance matrix

The measurement noise covariance matrix R has to be modified continuously. A particularly noticeable bias is introduced to lateral velocity and yaw rate measurements from the single track model etc. during hard cornering. Consequently, during non-linear vehicle behaviour, the measurement variances for the single cycle model should have very high values compared with the process noise values. This allows Kalman gain to weight more direct tyre force integration instead of obviously biased v_y_ and *ψ̇* measurements. A method to define measurement noise variance for single track model measurements is explained in the following.

#### 3.5.3. Evaluation of linearity of vehicle operating state

One way to evaluate whether the vehicle behaves as a linear system is to compare nominal yaw rate and actual yaw rate, which has been exploited in ESC-systems [1] and to estimate the friction coefficient [2]. However, if the tyre forces are available by measurement, it is natural to compare measured tyre forces with the single track model (nominal) tyre forces in order to evaluate whether the system is in a linear operating region:

(29) $$\Delta F_{y}=F_{y,f,\text{onetrack}}-F_{y,f,\text{measured}}|+\left|F_{y,r,\text{onetrack}}-F_{y,r,\text{measured}}\right|$$

**Figure 7.**
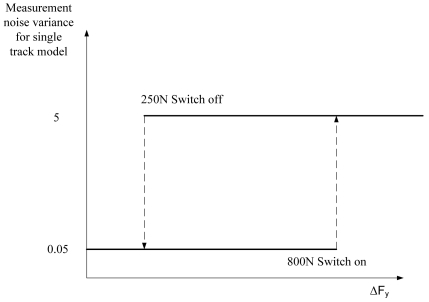
A relay with hysteresis to select proper measurement noise variance.

Depending on driving conditions, the corresponding measurement noise variance (for single track v_y_ and *ψ̇*) is selected based on ΔFy, where a relay with hysteresis is implemented (Figure 7). The tuning of the relay is essential to achieve a fast and stable response for the estimate. The low variance value can be tuned in straight ahead driving, where the variance can be adjusted to as great a value as the estimate vy without drifting, due to the integration of tyre forces. On the other hand, during cornering, the variance should switch to a noticeably higher level before the vehicle exhibits a non-linear operating region. The hysteresis is needed in order to avoid unnecessary relay switch offs, for example during lane changes, where single track model tyre forces and tyre sensor measurements can be almost equal temporarily.

Another method to judge vehicle non-linearity would be to evaluate the relation of lateral and vertical forces (Equation 14). However, the threshold value is difficult to determine because the cornering stiffness linearity region depends on road conditions.

## 4. Results

**Figure 8.**
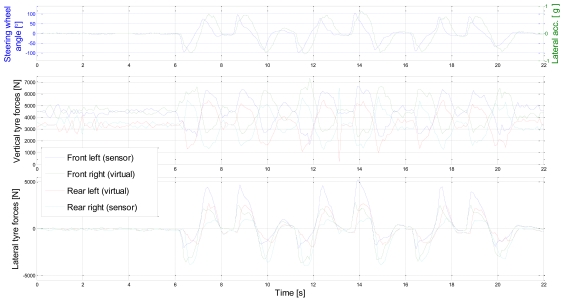
Lateral and vertical tyre forces for a driving manoeuvre (v_x_∼60 km/h, dry tarmac).

A test manoeuvre was sequential and aggressive lane changes on dry and horizontally even tarmac road. The lateral and vertical tyre forces for the test manoeuvre are shown in Figure 8. The influence of load transfer can be seen in the vertical forces. The vertical forces vary between 1,000 N to 7,000 N. For the lateral forces, peak values for the left front tyre sensor are actually overestimated, due to sensor-lens setup non-linearities, which were found at the edge of the operating area during high vertical force. This results in an overestimation of the normalised lateral force of the front axle; hence the right front tyre lateral force might be overestimated as well.

**Figure 9.**
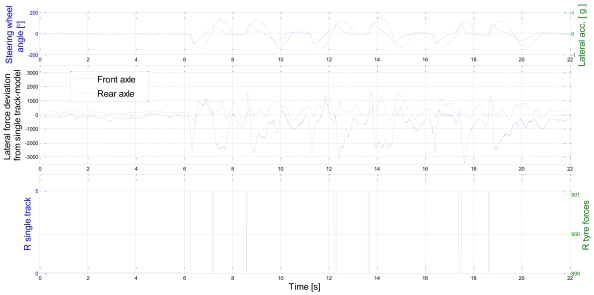
Lateral force deviation from single track model and measurement error variance during test run.

Figure 9 shows the lateral axle force deviation from the single track model as calculated in Equation 29 (the same test run as in Figure 8). This deviation is further on exploited to evaluate the validity of a single-track model state estimate. When the measured tyre forces deviate from the single track model estimate, the measurement noise variance of a single track model measurement is high. Note that the measurement noise variance for the tyre sensors is constant. This is realistic because the accuracy of the tyre sensor does not depend on driving conditions. The lowest plot shows how the relay with hysteresis operates according to the driving state. During lane change, short switch-offs are detected. Otherwise the relay can recognise the straight ahead driving and cornering.

**Figure 10.**
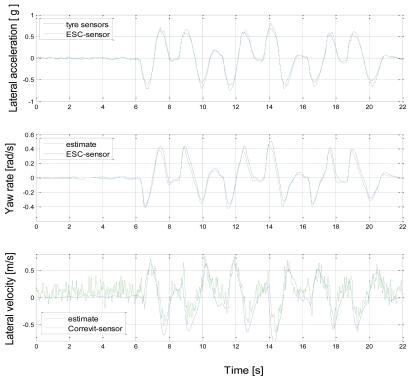
Lateral state estimate based Kalman filter estimator and for sensor measurement.

Figure 10 shows the lateral acceleration comparison with the measured (normalised) tyre forces. The correlation is rather good and slightly higher peak values for the lateral acceleration sensor might be influenced by roll angle (gravity component). The yaw rate comparison seems to produce slightly greater values for the estimate in general. The left front tyre estimate was observed to overestimate lateral force during high vertical forces; this can explain why the values were higher than expected during positive yaw rate (right turn). The underestimation of yaw rate for negative values is more difficult to explain, but the reasons may lie in the inaccuracy of the virtual tyre sensors or in toe-in and roll-steer induced offsets in lateral forces. The influence of front left tyre lateral force overestimation is then stronger for *ψ̇* than a_y_ (due to the integration step needed for *ψ̇*). Even if yaw rate estimate suffers from the explained inaccuracy in tyre force estimate, the overal performance for the lateral velocity estimate v_y_ remains tolerable. The yaw rate overestimation results in cumulation of error to the negative direction of v_y_.

## 5. Discussion

This paper presented one application for tyre sensors. The proposed vehicle lateral state estimator can similarly be used with other tyre force measurements than tyre sensors, such as suspension part or wheel hub strain measurements.

### 5.1. Can a tyre force sensor replace any of the existing vehicle sensors?

The main advantage of tyre force sensing is definitely the information given about the operating state of each tyre. In addition it is possible to calculate the lateral acceleration of a vehicle from the sum of tyre forces without any bias from body roll angle and road inclination. Also, the influence of side wind is realistically captured. The results show that lateral acceleration was accurately calculated from the tyre forces when measured by an optical tyre sensor. The yaw rate sensor, however, is much more complex to replace than the acceleration sensor. The required integration step makes the estimate extremely sensitive to errors in parameters l_f_ and l_r_, which may arise, for example, from the pneumatic trail in addition to the mechanical movement of the wheel hub. Thus, the yaw rate is a valuable measure of the differences in front and rear axle forces acting on a vehicle. However, if the tyre sensor can produce an accurate and reliable estimate for the vertical force, the vehicle centre of gravity position can be calculated in a steady state condition.

### 5.2. Required vehicle parameters by the estimator

One of the objectives of the estimator was to minimize the number of parameters. Table 1 presents required vehicle parameters by the estimator and proposes some possible sources for them.

The vehicle mass is naturally available from the vertical tyre forces, but it requires real force measurements instead of the virtual tyre sensors implemented in this paper. However, reasonable accuracy would also be possible with two tyre sensors.

### 5.3. Further research

Improvement of the optical tyre sensor operation region would enable accurate estimation of lateral force during high vertical force and high slip angle. The single track model could be extended to adapt the parameters to ensure accurate operation during low lateral excitation.

The main benefits of this proposed Kalman filter approach could be seen on slippery road conditions, on side wind, and on inclined roads, where the problems for the model based estimation based on non-linear vehicle model are seen. In addition, the tyre force based estimator can be fitted to totally new types of vehicles without any major parameter modifications as long as the axle length is known.

The virtual tyre sensor concept might be feasible together with a more production oriented tyre sensor, or other type of tyre force measurement. The virtual tyre sensor concept would also lower the threshold for production tyre sensors as half of the sensor costs would be saved if only two tyres of a car needed be equipped with tyre sensors. It is possible to do this research and development mainly with simulation models, with no significant investments needed for the tyre sensor prototypes. The main problems are the combined slip case and the slightly non-linear influence of the wheel load on tyre forces.

## Figures and Tables

**Table 1.** Required estimator parameters.

**Parameter**	**Definition**	**Source**	**Value**
m	Vehicle mass	available from vertical tyre forces	1,603 kg
l	axle length	vehicle parameter	2,575 m
l_f_	Centre of gravity distance from front axle	available from vertical tyre forces in steady state condition	1.05m
l_r_	Centre of gravity distance from rear axle	available from vertical tyre forces in steady state condition	1.525m
I_z_	Vehicle yaw moment of inertia	roughly *m·l_r_·l_r_* [24] or adapted	3,156 kg m^2^
Q	Process noise covariance	constant	diag([0.01 0.01 1e4 1e4])
R	Measurement noise covariance	derived in section 3.5	variable
C_f_ & C_r_	Cornering stiffness of the linear model (or characteristic velocity	ESC-system (nominal behaviour of a vehicle)	76,614 N/rad & 82,087N/rad
